# Predicting patient reported outcome measures: a scoping review for the artificial intelligence-guided patient preference predictor

**DOI:** 10.3389/frai.2024.1477447

**Published:** 2024-11-05

**Authors:** Jeremy A. Balch, A. Hayes Chatham, Philip K. W. Hong, Lauren Manganiello, Naveen Baskaran, Azra Bihorac, Benjamin Shickel, Ray E. Moseley, Tyler J. Loftus

**Affiliations:** ^1^Department of Surgery, University of Florida, Gainesville, FL, United States; ^2^Department of Health Outcomes and Biomedical Informatics, University of Florida, Gainesville, FL, United States; ^3^College of Medicine, University of Florida, Gainesville, FL, United States; ^4^Department of Medicine, University of Florida, Gainesville, FL, United States

**Keywords:** machine learn, palliative care, patient reported outcome, fairness, clinical decision support

## Abstract

**Background:**

The algorithmic patient preference predictor (PPP) has been proposed to aid in decision making for incapacitated patients in the absence of advanced directives. Ethical and legal challenges aside, multiple practical barriers exist for building a personalized PPP. Here, we examine previous work using machine learning to predict patient reported outcome measures (PROMs) for capacitated patients undergoing diverse procedures, therapies, and life events. Demonstrating robust performance in predicting PROMs for capacitated patients could suggest opportunities for developing a model tailored to incapacitated ones.

**Methods:**

We performed a scoping review of PubMed, Embase, and Scopus using the PRISMA-ScR guidelines to capture studies using machine learning to predict PROMs following a medical event alongside qualitative studies exploring a theoretical PPP.

**Results:**

Sixty-eight studies used machine learning to evaluate PROMs; an additional 20 studies focused on a theoretical PPP. For PROMs, orthopedic surgeries (*n* = 33) and spinal surgeries (*n* = 12) were the most common medical event. Studies used demographic (*n* = 30), pre-event PROMs (*n* = 52), comorbidities (*n* = 29), social determinants of health (*n* = 30), and intraoperative variables (*n* = 124) as predictors. Thirty-four different PROMs were used as the target outcome. Evaluation metrics varied by task, but performance was overall poor to moderate for the best reported scores. In models that used feature importance, pre-event PROMs were the most predictive of post-event PROMs. Fairness assessments were rare (*n* = 6). These findings reinforce the necessity of the integrating patient values and preferences, beyond demographic factors, to improve the development of personalized PPP models for incapacitated patients.

**Conclusion:**

The primary objective of a PPP is to estimate patient-reported quality of life following an intervention. Use of machine learning to predict PROMs for *capacitated* patients introduces challenges and opportunities for building a personalized PPP for *incapacitated* patients without advanced directives.

## Introduction

Machine learning and artificial intelligence-based algorithms are predicting our preferences on a daily basis. Using aggregated data from past actions—such as a purchase, click, or prolonged gaze—we are continuously offered things to buy, watch, and experience. In advertising, their accuracy can exceed 95% in some instances (Assistant et al., 2023). However, algorithmic preference predictors have not yet extended to the more somber, consequential domain of patient medical decision making.

The Patient Preference Predictor (PPP) for incapacitated patients has been debated in the literature for over 10 years (Rid, 2014). Defined as a tool to help clinicians and surrogate decision makers decided on life-sustaining treatment decisions, several authors have more recently proposed using artificial intelligence to gauge patients preferences when they are unable to make decisions for themselves (Biller-Andorno and Biller, 2019; Wendler et al., 2016). The psychosocial, ethical, and legal implications of using static, statistical evidence to predict end-of-life choices are substantial and complex. While it has been shown that they may provide a better indication of patient preferences than estranged family, friends, and court-designated surrogates–whose decisions are unfortunately often no better than chance (Rid and Wendler, 2010; Shalowitz et al., 2006)–these models would still leave myriad concerns related to loss of autonomy, fairness, lack of trust, and reproducibility (Rid, 2014; Jardas et al., 2021; Rid and Wendler, 2014a; Sharadin, 2018).

Many had hoped that widespread adoption of advanced directives would improve end-of-life decision making. Unfortunately, these documents, in addition to being sparsely available, are frequently too limited in scope for highly morbid interventions. They typically describe preferences for cardiopulmonary resuscitation (Do-Not-Resuscitate, DNR), intubation (Do-Not-Intubate, DNI), or hospitalization (Do-Not-Hospitalize, DNH), but fail to account for complex choices around feeding tube placement, prolonged mechanical ventilation, artificial cardiopulmonary support, or any procedure that leads to substantial change in quality of life (Fagerlin and Schneider, 2004; Detering et al., 2010). Moreover, patient preferences are protean. In the case of survival, they are subject to hindsight bias (Becerra Pérez et al., 2016), and in the case of death, are without a ground truth to know whether the patient received the care they wanted (Rid, 2014). The current practice is to hold the last stated desires as that ground truth (Rid, 2014; Jardas et al., 2021).

Artificial intelligence is currently being studied in thousands of predictive tasks in health care (Rajkomar et al., 2018; Rajpurkar et al., 2022). While these include complications and medical outcomes of interest, they are also increasingly focused on predicting Patient Reported Outcomes Measures (PROMs). PROMs reflect patient quality-of-life in a numeric form and may be a more personalized metric, unlike mortality or a complication defined by a diagnostic code (McGlothlin and Lewis, 2014; Weinfurt and Reeve, 2022). There is a small but rapidly growing interest in using pre-intervention variables, including quality-of-life metrics, to predict post-intervention patient perceptions of their care. PROMs are also expanding their presence in national databases, providing rich data sources for predictive tasks (Temple et al., 2024). We consider the PPP to be, at its core, a task of predicting patient-reported outcomes. Therefore, inclusion of PROMs for *capacitated* patients represent a potential ground truth for researchers interested in the feasibility and fairness of predicting preferences of *incapacitated* patients. In other words, if we know with reasonable certainty how a patient of certain characteristics and perceptions of their current quality of life would assess their life post-intervention, we can know whether or not they would prefer the intervention.

In this scoping review, we reconcile the philosophical and ethical debates of predicting *incapacitated* patient preferences with the current applications of machine learning in the real world for *capacitated* ones.

## Materials and methods

**Figure 1:**
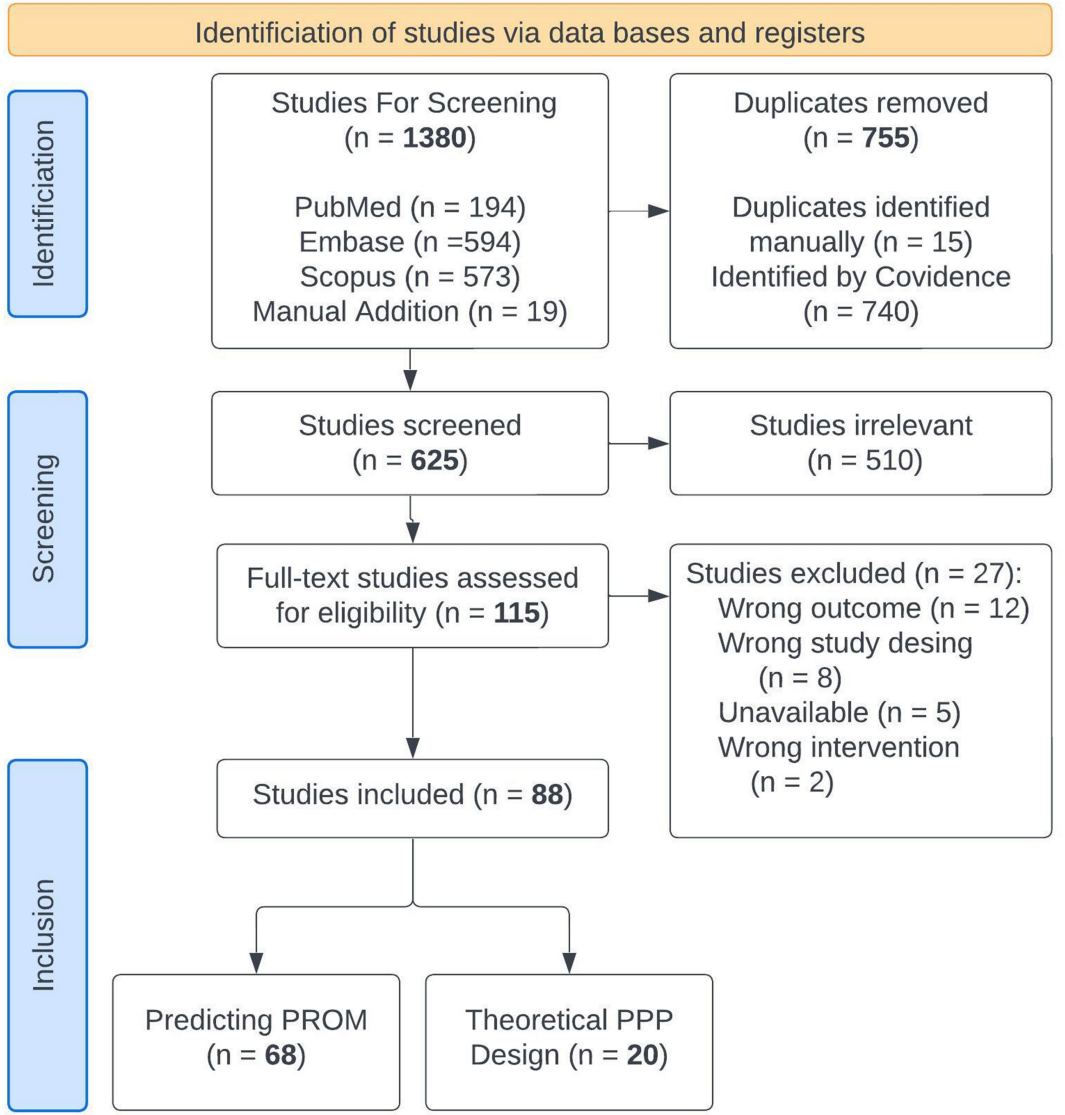
Article flow diagram.

We searched PubMed, Embase, and Scopus from January 1, 2019 to May 30, 2024 for terms related to machine learning for predicting PROMs to capture the most recent modeling techniques. Since PPPs for incapacitated patients are still theoretical, articles debating the ethical and practical issues of such models were reviewed separately. Search terms are shown in Supplementary file 1 and the PriSMA-ScR checklist is shown in Supplementary file 2. We identified 621 abstracts in the literature, which were reviewed by JAB, AHC, PH, LM, and NB. Cohen Kappa inter agreement scores ranged from 0.35–0.59. Disagreements were reviewed and resolved between the first author and the individual rater without need for arbitration. 115 full texts were reviewed by the first author. Twenty-seven studies were excluded leaving 88 studies for extraction. Eligible studies employed machine learning for a distinct, health-related event (surgical intervention, medical treatment, therapy secession, or diagnosis), and omitted post-event variables to predict the PROM in the outcome analysis. Twenty were theoretical discussions of patient preference predictors and 68 used machine learning to predict PROMs. Article flow is shown in Figure 1.

We extracted separate variables for the two sets of studies. For PROM studies, we gathered information on the study’s main findings, independent and dependent variables, data origin (intraoperative, in-patient, out-patient), data quality assessments, data source (single institution, multi-institution, national database, etc.), machine learning techniques, population characteristics, participant count, performance metrics, fairness metrics, and explainability techniques. Data quality was judged according to the TRIPOD+AI guidelines for machine learning tasks; studies were considered “excellent” if TRIPOD-AI or CONSORT-AI guidelines were followed, “good” if the methods described data preprocessing steps, handling missingness, and adjusting for class imbalance, and “fair” if they missed one or more of those qualities. Studies were excluded if methods failed to describe data cleaning, validation, and model development. For ethical studies, we performed a narrative thematic analysis for the ethical and legal principles identified, theoretical model inputs, fairness metrics, and proposed evaluation methods.

We employed Covidence® (Melbourne, Australia) software to manage multiple reviewers. Elicit® (Oakland, California) was used for initial data extraction, followed by manual confirmation and extraction of additional information (Elicit: The AI research Assistant, 2023).

## Results

### Study characteristics

**Figure 2:**
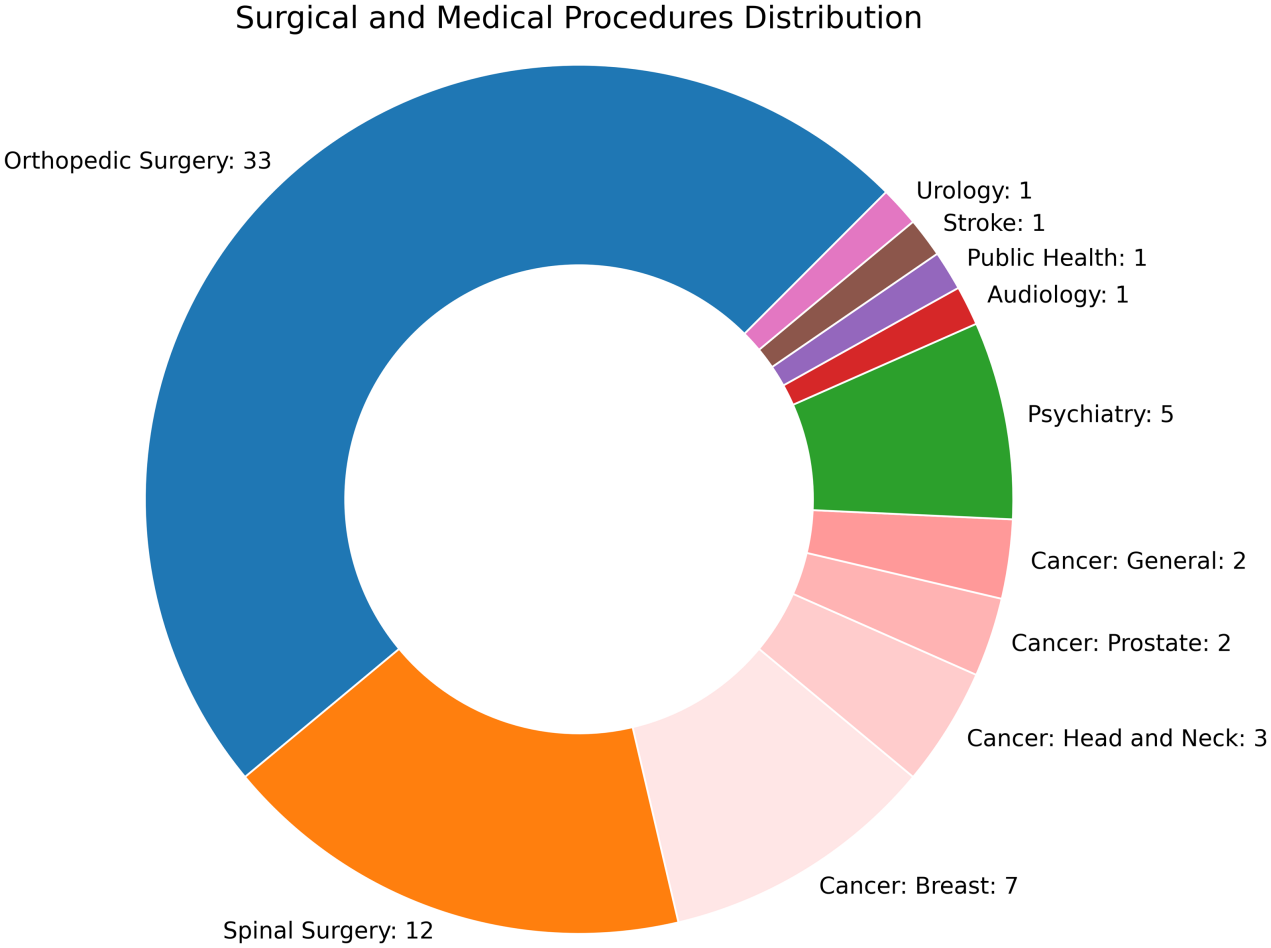
Distribution of studies by field.

Sixty-eight studies used machine learning to evaluate PROMs. All studies were retrospective, though three developed a web or smart phone based application (Karhade et al., 2021; Martin et al., 2022; Polce et al., 2021) and one study performed an external validation (Simmons et al., 2024). No studies examined how their findings altered clinical practice. The number of participants ranged from 22^23^ to 130,945 (Zrubka et al., 2022). Studies were performed either at a single hospital (*n* = 36), multiple hospitals (*n* = 23), or employed regional or national registries (*n* = 8). As shown in Figure 2, most studies were related to extremity orthopedic surgeries (*n* = 33) or spinal surgeries (*n* = 11), followed by oncology (*n* = 8 for breast; *n* = 7 for head and neck, prostate, and general), and psychotherapy (*n* = 5). Clinical events for before and after comparisons included invasive procedures (*n* = 48), medical and psychological therapy (*n* = 7), diagnoses (*n* = 6), physical therapy (*n* = 2), and a medical device (*n* = 1), with some studies examining surgical and adjuvant therapy for cancer (*n* = 3). A substantial amount of research has been performed in predicting PROMs following total knee arthroplasty (TKA), with 17 studies examining this question alone and an additional 13 examining other extremity joint surgeries. 22% (*n* = 15) followed either the TRIPOD-AI or CONSORT-AI guidelines, 35.3% (*n* = 24) were ranked as “good,” and 41.2% (*n* = 28) were “fair.”

### Outcomes

Over a dozen PROM instruments were found in this literature review and are listed in Table 1. No one score predominated. The orthopedic studies focused on validated orthopedic PROM metrics. These include the ASES and ESES scores (American and European Shoulder and Elbow Surgeons) (Kumar et al., 2020; Alaiti et al., 2023; Taneja et al., 2024), COMI (Core Outcome Measures Index) (Halicka et al., 2023), Global Perceived Effect (Verma et al., 2023; Verma et al., 2022), KOOS (Knee Injury and Osteoarthritis Outcome Score) (Martin et al., 2022; Harris et al., 2021; Katakam et al., 2022; Ramkumar et al., 2021; Klemt et al., 2023; Fontana et al., 2019; Twiggs et al., 2021), Lysholm functional protocol (Ye et al., 2022), Oswestry Disability Index (Staartjes et al., 2019; Siccoli et al., 2019), QuickDASH (Quick Disabilities of the Arm, Shoulder, and Hand) (Brinkman et al., 2023; Harrison et al., 2022), Oswestry Disability Index (Staartjes et al., 2019), iHOT (International Hip Outcome Tool) (Pettit et al., 2023), HOS (Hip Outcome Score) (Kunze et al., 2021), HOOS (Hip Disability and Osteoarthritis Outcome Score) (Klemt et al., 2023; Fontana et al., 2019; Sniderman et al., 2021), IKDC (International Knee Documentation Committee) (Ramkumar et al., 2021; Ye et al., 2022; Ramkumar et al., 2021), MHQ (Michigan Hand outcomes Questionnaire) (Loos et al., 2022), Q score (Oxford Hip and Knee Score) (Huber et al., 2019), SRS-22r (Sociolois Research Society) (Ames et al., 2019; Nnamdi et al., 2023), and the WOMAC (Western Ontario and McMaster Universities Osteoarthritis Index) (Munn et al., 2022; Tschuggnall et al., 2021; Zhang et al., 2022; Zhou et al., 2023). These scores capture pain, symptoms, mobility/functionality, activities of daily living, and quality of life metrics related to the joint of interest, including the spine. Cancer-related tools included the BREAST-Q (Pfob et al., 2021; Pfob et al., 2023; Xu et al., 2023), Cancer Related Fatigue (Beenhakker et al., 2023), Lee Fatigue Scale (Kober et al., 2023; Kober et al., 2021), EORTC QLQ-C3 (European Organization for Research and Treatment of Cancer quality-of-life questionnaires) (Lee et al., 2020a), MDADI (MD Anderson Dysphagia Inventory) (Paetkau et al., 2024), IPSS (International Prostate Symptom Score) (Ghoreifi et al., 2023), EPIC 26 (Expanded Prostate Cancer Index Composite 26) (Agochukwu-Mmonu et al., 2022), and THYCA-QoL (Thyroid Cancer Quality of Life) (Lian et al., 2023). These score focus on measures related to symptoms following treatment, such as mastectomy results, fatigue, dry mouth, erectile dysfunction, etc. More universal quality-of-life PROMs included instruments and sub-instruments of the COST (COmprehensive Score for financial Toxicity) (Sidey-Gibbons et al., 2021), HAQ (Health Assessment Questionnaire) (Tschuggnall et al., 2021), EQ-5D-3L (EuroQol 5-Dimension 3-Level) (Zrubka et al., 2022; Harrison et al., 2022; Huber et al., 2019; Tschuggnall et al., 2021), PHQ-9 (Patient Health Questionnaire-9) (Coley et al., 2021; Bone et al., 2021), PASS (Patient Acceptable Symptom State) (Twiggs et al., 2021), PROMIS (Patient-Reported Outcomes Measurement Information System) (Karhade et al., 2021; Klemt et al., 2023; Brinkman et al., 2023; Hunter et al., 2024; Reps et al., 2022), and several versions of SF (Short Form Survey) (Ramkumar et al., 2021; Fontana et al., 2019; Ramkumar et al., 2021; Munn et al., 2022; Zhang et al., 2022; Zhou et al., 2023; Lian et al., 2023). In addition, several studies employed more basic instruments, capturing Visual Analogue Scores of Pain (Kumar et al., 2020; Halicka et al., 2023; Harris et al., 2021; Staartjes et al., 2019; Dolendo et al., 2022; Finkelstein et al., 2021; Park et al., 2023), numeric pain scores (Siccoli et al., 2019), and patient satisfaction scores on a Likert scale (Polce et al., 2021; Kumar et al., 2020; Munn et al., 2022; Farooq et al., 2020; Kunze et al., 2021; Kunze et al., 2020; Nam et al., 2023; Ulivi et al., 2023; Wang et al., 2023; Werneburg et al., 2023). Psychological measurements included GAD-7 (Generalized Anxiety Disorder) (Bone et al., 2021; Reps et al., 2022). One studied used the COSI (Client Oriented Scale of Improvement) for audiology (Suresh et al., 2023). Overall, these scores capture both cognitive, pain-related, and functional aspects of quality of life. 20 studies used minimally clinical important difference (MCID) on before and after scores of the PROMs to create a binary classification task.

**Table 1:** Patient reported outcome measure (PROM) instruments.

Orthopedic PROM	
ASES and ESES scores (American and European shoulder and elbow surgeons) (Kumar et al., 2020; Alaiti et al., 2023; Taneja et al., 2024) COMI (Core outcome measures index) (Halicka et al., 2023) Global perceived effect (Verma et al., 2023; Verma et al., 2022) KOOS (Knee injury and osteoarthritis outcome score) (Martin et al., 2022; Harris et al., 2021; Katakam et al., 2022; Ramkumar et al., 2021; Klemt et al., 2023; Fontana et al., 2019; Twiggs et al., 2021) Lysholm functional protocol (Ye et al., 2022) Oswestry disability index (Staartjes et al., 2019; Siccoli et al., 2019) QuickDASH (Quick disabilities of the arm, shoulder, and hand) (Brinkman et al., 2023; Harrison et al., 2022) Oswestry disability index (Staartjes et al., 2019)	iHOT (International hip outcome tool) (Pettit et al., 2023) HOS (Hip outcome score) (Kunze et al., 2021) HOOS (Hip disability and osteoarthritis outcome score) (Klemt et al., 2023; Fontana et al., 2019; Sniderman et al., 2021) IKDC (International knee documentation committee) (Ramkumar et al., 2021; Ye et al., 2022; Ramkumar et al., 2021) MHQ (Michigan hand outcomes questionnaire) (Loos et al., 2022) Q score (Oxford hip and knee score) (Huber et al., 2019) SRS-22r (Sociolois research society) (Ames et al., 2019; Nnamdi et al., 2023) WOMAC (Western ontario and mcmaster universities osteoarthritis index) (Munn et al., 2022; Tschuggnall et al., 2021; Zhang et al., 2022; Zhou et al., 2023)
Oncologic PROM	
BREAST-Q (Pfob et al., 2021; Pfob et al., 2023; Xu et al., 2023) Cancer related fatigue (Beenhakker et al., 2023) Lee Fatigue Scale (Kober et al., 2023; Kober et al., 2021) EORTC QLQ-C3 (European organization for research and treatment of cancer quality-of-life questionnaires) (Lee et al., 2020a; Lee et al., 2020b)	MDADI (MD Anderson dysphagia inventory) (Paetkau et al., 2024) IPSS (International prostate symptom score) (Ghoreifi et al., 2023) EPIC 26 (Expanded prostate cancer index composite 26) (Agochukwu-Mmonu et al., 2022) and THYCA-QoL (Thyroid cancer quality of life) (Lian et al., 2023)
General PROM	
COST (Comprehensive score for financial Toxicity) (Sidey-Gibbons et al., 2021) HAQ (Health assessment Questionnaire) (Tschuggnall et al., 2021) EQ-5D-3 L (EuroQol 5-dimension 3-Level) (Zrubka et al., 2022; Harrison et al., 2022; Huber et al., 2019; Tschuggnall et al., 2021) PHQ-9 (Patient health questionnaire-9) (Coley et al., 2021; Bone et al., 2021)	PASS (Patient acceptable symptom state) (Twiggs et al., 2021) PROMIS (Patient-reported outcomes measurement information system) (Karhade et al., 2021; Klemt et al., 2023; Brinkman et al., 2023; Hunter et al., 2024; Reps et al., 2022) SF (Short form survey) (Ramkumar et al., 2021; Fontana et al., 2019; Ramkumar et al., 2021; Munn et al., 2022; Zhang et al., 2022; Zhou et al., 2023; Lian et al., 2023) GAD-7 (Generalized anxiety disorder) (Bone et al., 2021; Reps et al., 2022)
Pain, visual analogue score (Kumar et al., 2020; Halicka et al., 2023; Harris et al., 2021; Staartjes et al., 2019; Dolendo et al., 2022; Finkelstein et al., 2021; Park et al., 2023)	
Patient satisfaction scores, Likert scale (Polce et al., 2021; Kumar et al., 2020; Munn et al., 2022; Farooq et al., 2020; Kunze et al., 2021; Kunze et al., 2020; Nam et al., 2023; Ulivi et al., 2023; Wang et al., 2023; Werneburg et al., 2023)	

### Input data

**Figure 3:**
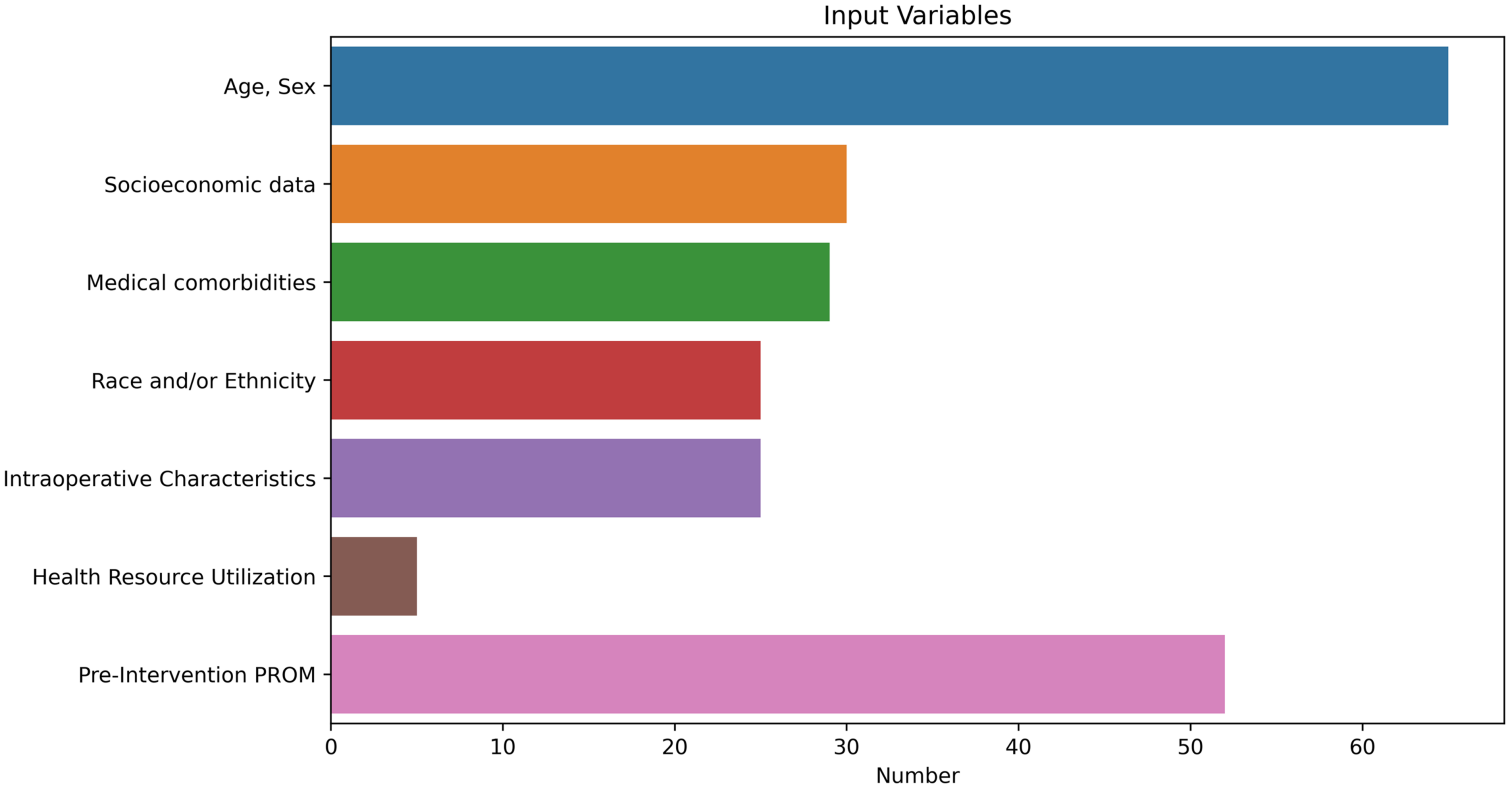
Input variables for patient reported outcome measure (PROM) prediction tasks.

Nearly all studies included demographic data as model features (*n* = 65). The three remaining studies examined unstructured text (Lian et al., 2023; Wang et al., 2023; Matsuda et al., 2023). Twenty-nine studies included medical comorbidities. Thirty studies included sociodemographic data, including marital status, employment status, insurance information, drug use, and zip-code level income and education indices. Five studies assessed health care resource utilization, including hospitalizations and emergency room visits. Twenty-three of 36 studies involving surgeries included intraoperative characteristics, including surgeon, technical approach, types of implants used, characteristics of the tumor, and operative time. Fifty-two studies employed pre-event PROMs and included both the PROM outcome of interest alongside addition PROM metrics. Proportions of input variables are visualized in Figure 3.

### Machine learning models

**Figure 4:**
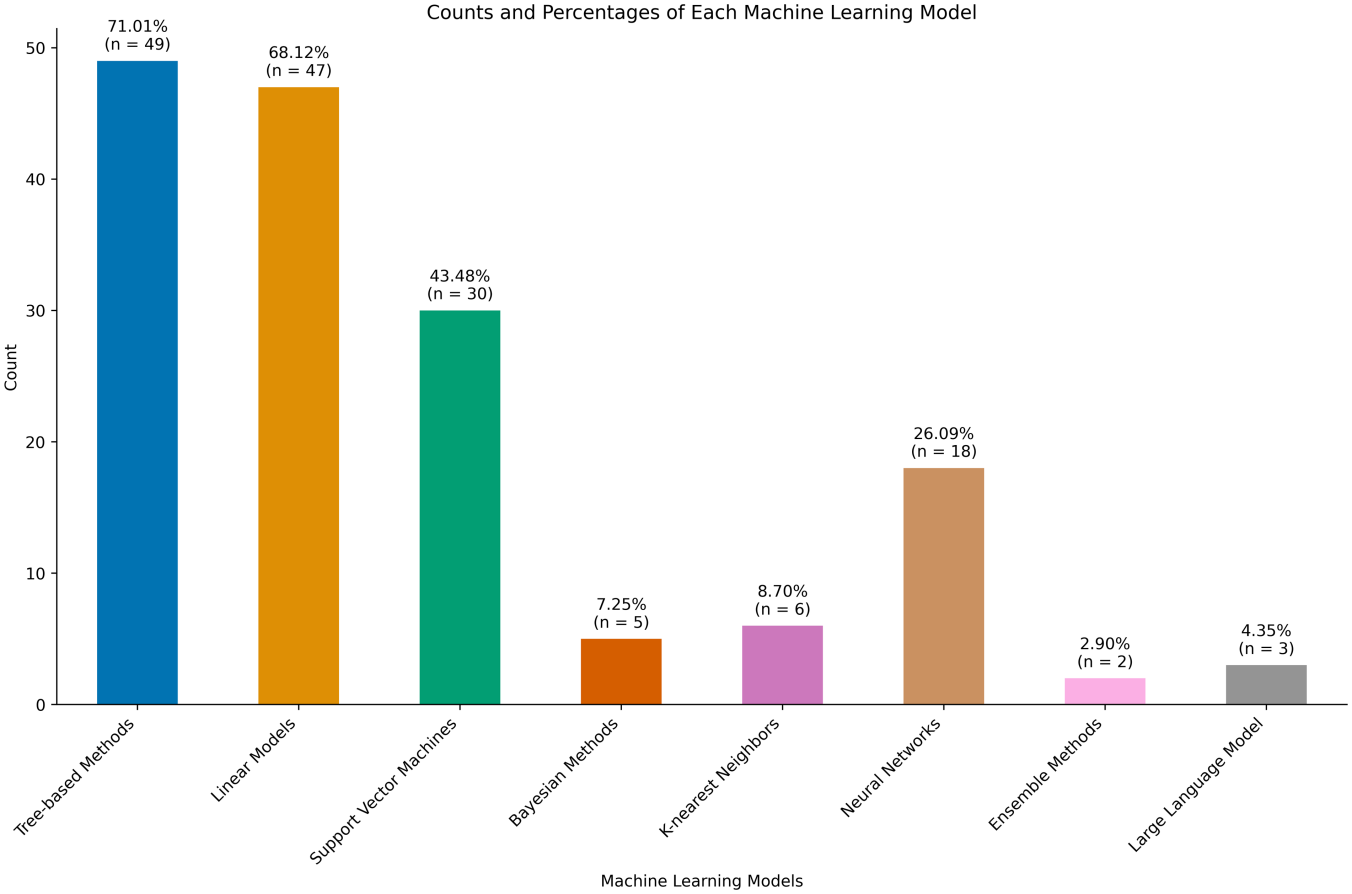
Machine learning models employed for patient reported outcome measure (PROM) prediction tasks.

A range of machine learning techniques were employed. The majority (*n* = 47) included some logistic or linear regression task as a base of comparison. All common machine learning models were employed, including linear and logistic regression, naive bayes, support vector machines, decision trees, random forest, and ensemble methods. These are shown in Figure 4. Only three studies employed large language models, both using the Bidirectional Encoder Representations from Transformers (BERT) architecture (Lian et al., 2023; Wang et al., 2023; Matsuda et al., 2023). Of note, 50 studies had some mention of data quality assessment. The majority addressed methods for handling missing data, largely through imputation or exclusion. Ten studies mentioned methods of handling class imbalance (Alaiti et al., 2023; Taneja et al., 2024; Ramkumar et al., 2021; Staartjes et al., 2019; Siccoli et al., 2019; Ramkumar et al., 2021; Ames et al., 2019; Zhang et al., 2022; Zhang et al., 2021; Chen et al., 2023).

### Evaluation metrics

**Figure 5:**
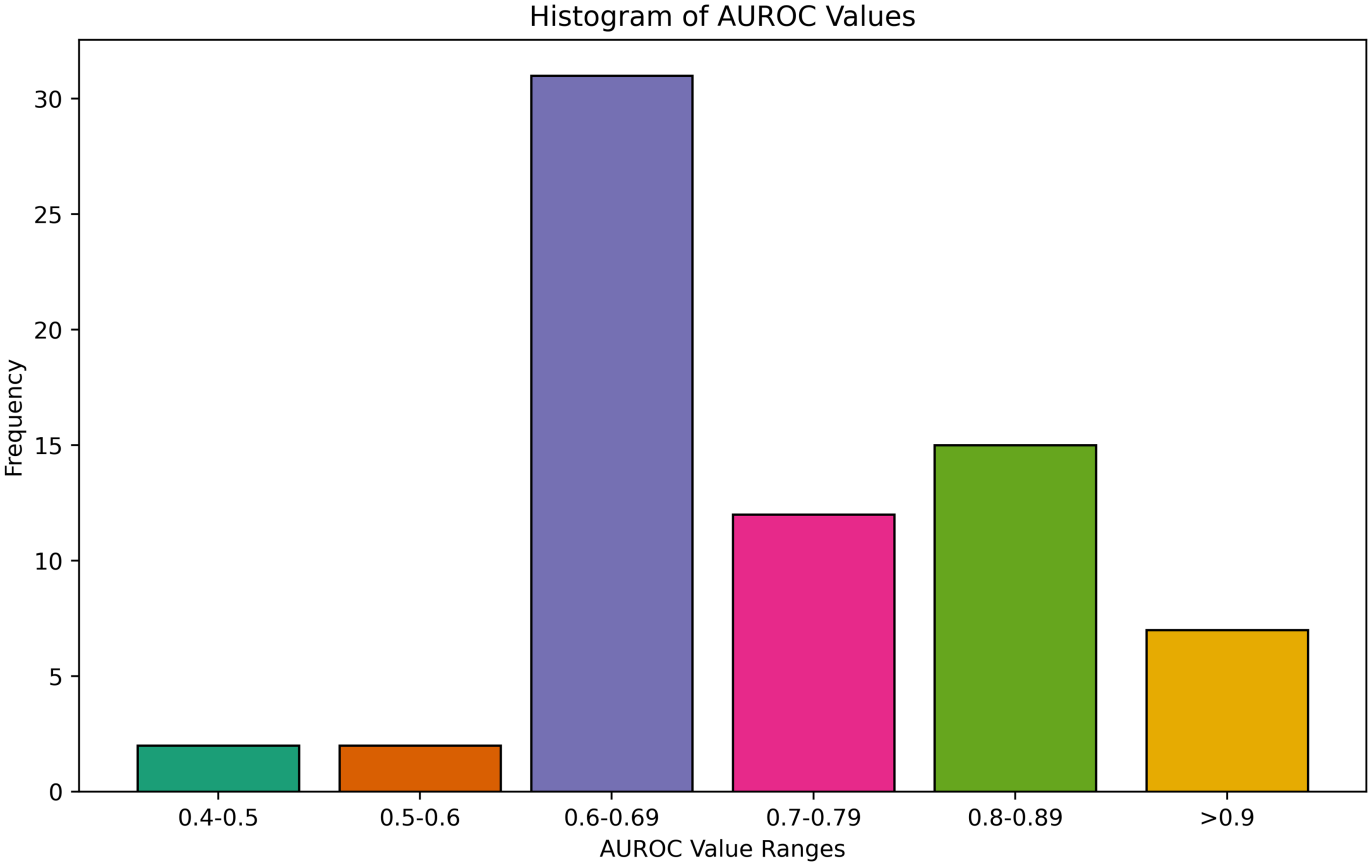
Area under the receiver operating curve (AUROC) performance metric distribution.

Overall, models performed poorly or moderately well, with few models approaching excellent discriminative capacity of AUROC exceeding 0.9. AUROC results ranged from 0.42 to 0.94 for any binary prediction task, with a mean of 0.78 and median of 0.77 among all best-performing AUROCs reported. Several studies concluded that no meaningful relationship exists between pre-event variables and PROMs in their feature space, suggesting a need to collect more data or different variables (Ghoreifi et al., 2023; Halicka et al., 2023; Verma et al., 2023; Pettit et al., 2023; Loos et al., 2022; Beenhakker et al., 2023; Coley et al., 2021; Ulivi et al., 2023). A histogram of performance is shown in Figure 5. A few studies, however, found high discriminative performance, including for predicting MCID for improvement in back pain following lumbar disectomy (Staartjes et al., 2019) and hip pain following total hip arthroplasty (Kunze et al., 2021) as well as satisfaction with outcomes following mastectomy for cancer (Pfob et al., 2021). Other evaluation metrics included MSE and R (Rid, 2014), again with moderate performance at best (Ghoreifi et al., 2023; Verma et al., 2022; Agochukwu-Mmonu et al., 2022; Finkelstein et al., 2021; Ulivi et al., 2023; Suresh et al., 2023). There was no association between model type and performance. We also assessed calibration, which quantifies how much a model over or underestimates the probability of an event, an often overlooked, but no less important, metric (van den Goorbergh et al., 2022; Van Calster et al., 2019). 35.3% (*n* = 24) of studies evaluated the calibration of their models. The calibration was overall good, with excellent calibration metrics (intercepts ≤ ± 0.1 and slopes between 0.9 to 1.1) in 21% (4/19) of models that reported intercept and slope (Karhade et al., 2021; Halicka et al., 2023; Agochukwu-Mmonu et al., 2022; Ziobrowski et al., 2021). Other papers used Brier (Harris et al., 2021; Siccoli et al., 2019), Hosmer-Lemeshow (Martin et al., 2022; Lee et al., 2020a), and Speigelhatler (Xu et al., 2023) tests to prove calibration, noting acceptable performance.

### Fairness and importance testing

While almost all models collected demographic information and mentioned need for external validation as a limitation to their generalizability, only six studies (8.9%) explicitly mentioned fairness or methods to mitigate bias (Simmons et al., 2024; Zrubka et al., 2022; Pfob et al., 2021; Pfob et al., 2023; Xu et al., 2023; Ziobrowski et al., 2021). Ziobrowski et al. examined model performance across age, sex, race/ethnicity, and income by estimating variations in the association of predicted risk with observed outcome using robust Poisson regression (Ziobrowski et al., 2021). In both their studies, Pfob et al. tested their models with and without sociodemographic and ethnic variables (fairness through unawareness) and obtained similar model performance (Pfob et al., 2021; Pfob et al., 2023). Zruboka, Simmons, and Xu evaluated prediction errors across different health statuses and demographics according to the PROM, with only the latter finding improve statistical performance for the African American group (Simmons et al., 2024; Zrubka et al., 2022; Xu et al., 2023). Simmons used the “four-fifths” legal guideline from the US Equal Employment Opportunity Commission to state that a “fair” model performs within 20% on any evaluation metric between demographic groups (Simmons et al., 2024). They found that ethnicity was rarely, but most frequently, outside this tolerance threshold, which the authors attributed to under-representation in the dataset.

Several studies employed potential fairness mitigation efforts without clear mention. One study employed inverse probability weighting to minimize the effects of missing data or under-represented groups, a potential marker of fairness but one that was not explicitly stated (Martin et al., 2022). Synthetic Minority Oversampling Technique (SMOTE) creates artificial data points that are plausibly close to actual data points and can be used as a fairness technique (Zhou et al., 2023). Several studies employed SMOTE create more balanced datasets in terms of their outcome of interest, and while this theoretically may improve representation of other minority classes, no study specifically examined this. However, we note that changing the overall prevalence of data classes through synthetic means may negatively impact model calibration (van den Goorbergh et al., 2022).

Importance testing was performed in 50 studies. Where performed, pre-event PROMs were either the largest or second largest contributor of post-event PROMs in all model predictions (Polce et al., 2021; Zrubka et al., 2022; Verma et al., 2023; Staartjes et al., 2019; Pettit et al., 2023; Munn et al., 2022; Pfob et al., 2021; Pfob et al., 2023; Xu et al., 2023; Kober et al., 2023; Kober et al., 2021; Park et al., 2023; Nam et al., 2023; Ulivi et al., 2023; Zhang et al., 2021). However, the correlations were not necessarily directly proportional: strong negative correlations of low PROMs sometimes predicted larger improvements in orthopedic studies, while other times demonstrated that PROMS in mobility, satisfaction rates, and narcotic use are unchanged after an event. Other top features trailed the PROMs, but included age, sex, BMI, patient anatomy, and comorbidities. Except for one study examining financial toxicity, where African American race was found to be predictive of toxicity (Sidey-Gibbons et al., 2021), no studies that measured ethnic or socioeconomic information reported its appearance in the top 5 predictive factors.

### Narrative thematic analysis of theoretical PPPs

Patient preference predictors have been discussed in the literature since a series of publications in the *Journal of Medicine and Philosophy* in 2014 (Rid, 2014; Rid and Wendler, 2014a; Kim, 2014; Rid and Wendler, 2014b). With the growing prevalence of machine learning in medicine, the issue was re-visited in second series in 2022 in the *Journal of Medical Ethics* (Jardas et al., 2021; Earp, 2022; Ferrario et al., 2022; Schwan, 2022; Mainz, 2023). As technologies advance, the debates are becoming increasingly pertinent. In our analysis, we address key themes such as ethical considerations, the selection of model inputs, fairness in predictions, and the evaluation of model efficacy.

#### Ethical considerations

Patient autonomy is of utmost concern in the PPP, however, autonomy can be defined in both a primary sense (“I would not want CPR done”) as well as a higher-order sense (“A decision was made for reasons I do not endorse”) (Earp, 2022). Identifying what an incapacitated patient would want might also involve knowledge of how they prefer to make decisions. A second concern is the legal problem of using “naked statistical evidence.” (Sharadin, 2018; Earp, 2022; Mainz, 2023; Ditto and Clark, 2014) Legal verdicts cannot be based on statistical correlations alone, as they do not imply causation, and the same may be said for a PPP. A third involves the lack of explainability with erosion of trust (Ferrario et al., 2022) mandating an alternative to “black box” models. Fourth, there potential for conflicting outputs by different PPPs (Sharadin, 2018) and even whether or not the patient would consent to the use of a PPP (Mainz, 2023). Deployment of these models would require extensive generalizability testing and buy-in from the public. Nevertheless, these articles acknowledge that a theoretical PPP has a low bar for improving decision making for incapacitated patients: human surrogate decisions, when analyzed retrospectively, are only slightly better than chance (Rid and Wendler, 2010; Shalowitz et al., 2006).

#### Model inputs

The discussions are fairly similar in their desired inputs for such a model and include: demographics, religious affiliations (Jardas et al., 2021; Rid and Wendler, 2014a; Sharadin, 2018; Rid and Wendler, 2014b; Earp, 2022; Ditto and Clark, 2014; Earp et al., 2024), level of risk taking (Jardas et al., 2021; Ditto and Clark, 2014), past treatment decisions (Rid and Wendler, 2014b; Mainz, 2023; Ditto and Clark, 2014; Earp et al., 2024; Benzinger et al., 2023), and baseline comorbidities (Mainz, 2023; Ditto and Clark, 2014). However, others call for more detailed examinations of attitudes toward death (Rid and Wendler, 2014a), personal experience with health care (Rid and Wendler, 2014a; Earp et al., 2024), and psychological and emotional functioning (Rid and Wendler, 2014a). Several argue for nation-level surveys to assess preferences and build more accurate models (Rid and Wendler, 2014a; Kim, 2014; Ditto and Clark, 2014), design forecasting scenarios of possible treatment outcomes (Ferrario et al., 2022), or even scraping publicly available information (Earp et al., 2024). As these studies focused more broadly on end-of-life decisions and not on specific operations or outcomes, none suggested intraoperative details or patient anatomy as a predictive measure.

#### Fairness

Model inputs are driven by the desire to build not only accurate models, but fair and just ones. Several papers warn that AI models may perpetuate social injustice (Rid, 2014; Biller-Andorno and Biller, 2019; Ferrario et al., 2022; Benzinger et al., 2023). In addition to incorporating various demographic and socioeconomic features, the perspectives of both the ill and the healthy must be incorporated to not unduly bias models toward one class of patients over another (Rid and Wendler, 2014b). Additionally, several authors mention that the just models would likely have to also understand what variables matter to the patient, i.e., whether or not to include religion, race, or education level as a factor (Wendler et al., 2016; Sharadin, 2018; Ferrario et al., 2022; Mainz, 2023). PROMs may potentially capture this variability, as they reflect direct, subjective patient expressions of their well-being. However, PROMs are not directly discussed by any of the cited articles.

#### Evaluation

Curiously, how to evaluate the accuracy of such models is also not often discussed (Rid and Wendler, 2014a). Many authors assume that surveying patients and their family members regarding decisions in hypothetical cases is sufficient to determine the accuracy of such models, however, given that patient preferences can often change radically in response to illness and end-of-life events, we ultimately lack a ground truth once incapacity has occurred. We know that interviewing survivors introduces a hindsight bias in treatment and that patients experience regret in only a minority of cases (Becerra Pérez et al., 2016; Rid and Wendler, 2014b). While several de-biasing strategies exist, no studies of predicting PROMs adjusted for hindsight bias in their analysis (Roese and Vohs, 2012).

Importantly, nearly all studies cautioned that the use of a patient preference predictors should only complement, and never replace, the provider or surrogate in making decisions for patients.

## Discussion

This scoping review takes a novel approach to the theoretical development of the fair patient preference predictor but hypothesizing that the PPP would function essentially as PROM predictor. We show how current machine learning techniques predict PROMs for capacitated patient undergoing healthcare-related interventions might translate to predicting PROMs as a surrogate metric for incapacitated patient. We show that models had poor to moderate performance in predicting PROMs, the most important input variables were often from a pre-event PROM survey, and that few investigators directly assessed the fairness of their models.

There has been one previous review of using machine learning to predict PROMs (Verma et al., 2021) and another has called for placing them at the forefront of clinical AI research (Cruz Rivera et al., 2023). This has several implications to building the patient preference predictor. First, we see that demographics, social determinants, and even medical comorbidities rarely feature in the top 10 feature importance graphs, despite their inclusion in the majority of studies. Second, we see that baseline surveys of pain, functionality, and satisfaction are highly correlated with future PROMs. Third, fairness assessments on sociodemographic variables were rare, but when performed, were often reassuring. Given that sociodemographic variables were less predictive than pre-intervention PROM scores, it is possible that building a patient preference predictor incorporating these variables of functionality and wellbeing would be fair. This, however, does not negate the need the perform fairness testing. Fourth, we find a robust system of measuring patient satisfaction in place for select medical subspecialties (orthopedics). Documenting before and after changes in PROMs to establish MCID may benefit the future development of a patient preference predictor. We see promising developments with the incorporation of PROMs into the National Surgical Quality Improvement Program (NSQIP) (Temple et al., 2024). Finally, we find that large language models are showing potential for extracting this kind of information from unstructured textual data (Lian et al., 2023; Wang et al., 2023; Matsuda et al., 2023).

We noted several limitations to the included studies. Existing models have overall small numbers compared to the thousands to millions of examples machine learning models benefit from. This limits their generalizability but also highlights the difficulty of collecting quality of life metrics on patients, which are unfortunately limited to burdensome survey or interview data. Likely because of this, model performance is poor to moderate with AUROC’s rarely exceeding 0.90. Second, nearly half of the studies were focused on extremity joint surgeries, which may limit generalizability, but remains informative based on the individual study’s choice of model inputs, architectures, and evaluation metrics. Third, we note the wide variety of PROM metrics used. While these are helpful to hyper-specific outcomes, we would like to see more generalizable and wildly used PROM metrics to facilitate generalizability. Fourth, few studies report evaluation metrics outside AUROC, including AUPRC and F1 scores, which may be better at capturing rare events. It is up to the individual specialty to determine the appropriate threshold for clinical use, but models that aid in predicting life and death decisions for incapacitated patients would likely require a higher bar.

## Conclusion

This review highlights many of the issues discussed in machine learning predictions of patient-centered outcomes. There are numerous practical, legal, and ethical barriers to using statistical evidence to fairly anticipate a decision in the incapacitated patient. Although machine learning models typically have poor to moderate performance in predicting PROMs, they often compare favorably with human surrogate decisions, which are only slightly better than chance.
