# Supplementary material for: Predicting patient reported outcome measures: a scoping review for the artificial intelligence-guided patient preference predictor
Source: Front Artif Intell. 2024 Nov 5;7:1477447. doi: 10.3389/frai.2024.1477447 (PMC11573790; doi:10.3389/frai.2024.1477447)
Supplement: Supplementary file 1 [file Table_1.DOCX]

Supplemental Digital Content 1 – Search Terms

PubMed

("patient preference predict*") OR ("patient decision" AND "predict*") OR ("patient satisfaction" AND "predict*") OR ("patient-reported outcomes" AND "predict*") AND ("machine learning" OR "artificial intelligence")

Scopus

(TITLE-ABS-KEY("patient preference predict*") OR TITLE-ABS-KEY("patient decision" AND predict*) OR TITLE-ABS-KEY("patient satisfaction" AND predict*) OR TITLE-ABS-KEY("patient-reported outcomes" AND predict*)) AND (TITLE-ABS-KEY("machine learning") OR TITLE-ABS-KEY("artificial intelligence"))

Embase

('patient preference predict*' OR ('patient decision' AND predict*) OR ('patient satisfaction' AND predict*) OR ('patient-reported outcomes' AND predict*)) AND ('machine learning' OR 'artificial intelligence')
